# Chemical defense of an Asian snake reflects local availability of toxic prey and hatchling diet

**DOI:** 10.1111/jzo.12004

**Published:** 2012-12-17

**Authors:** D A Hutchinson, A H Savitzky, G M Burghardt, C Nguyen, J Meinwald, F C Schroeder, A Mori

**Affiliations:** 1 Department of Biology, Coastal Carolina UniversityConway, SC, USA; 2 Department of Biological Sciences, Old Dominion UniversityNorfolk, VA, USA; 3 Department of Biology, Utah State UniversityLogan, UT, USA; 4 Departments of Psychology and Ecology & Evolutionary Biology, University of TennesseeKnoxville, TN, USA; 5 Department of Chemistry and Chemical Biology, Cornell UniversityIthaca, NY, USA; 6 Boyce Thompson Institute, Cornell UniversityIthaca, NY, USA; 7 Department of Zoology, Graduate School of Science, Kyoto UniversitySakyo, Kyoto, Japan

**Keywords:** *Rhabdophis*, *Bufo*, bufadienolides, nuchal glands, dietary toxin sequestration

## Abstract

Species that sequester toxins from prey for their own defense against predators may exhibit population-level variation in their chemical arsenal that reflects the availability of chemically defended prey in their habitat. *Rhabdophis tigrinus* is an Asian snake that possesses defensive glands in the skin of its neck (‘nuchal glands’), which typically contain toxic bufadienolide steroids that the snakes sequester from consumed toads. In this study, we compared the chemistry of the nuchal gland fluid of *R. tigrinus* from toad-rich and toad-free islands in Japan and determined the effect of diet on the nuchal gland constituents. Our findings demonstrate that captive-hatched juveniles from toad-rich Ishima Island that had not been fed toads possess defensive bufadienolides in their nuchal glands, presumably due to maternal provisioning of these sequestered compounds. Wild-caught juveniles from Ishima possess large quantities of bufadienolides, which could result from a combination of maternal provisioning and sequestration of these defensive compounds from consumed toads. Interestingly, juvenile females from Ishima possess larger quantities of bufadienolides than do juvenile males, whereas a small sample of field-collected snakes suggests that adult males contain larger quantities of bufadienolides than do adult females. Captive-born hatchlings from Kinkasan Island lack bufadienolides in their nuchal glands, reflecting the absence of toads on that island, but they can sequester bufadienolides by feeding on toads (*Bufo japonicus*) in captivity. The presence of large quantities of bufadienolides in the nuchal glands of *R. tigrinus* from Ishima may reduce the risk of predation by providing an effective chemical defense, whereas snakes on Kinkasan may experience increased predation due to the lack of defensive compounds in their nuchal glands.

## Introduction

Animals that rely on chemicals for antipredator defense may synthesize those compounds from nontoxic precursors (Daly, 1995; Eisner, Eisner & Siegler, 2005) or sequester defensive toxins from other organisms (González, Hare & Eisner, 1999; Nishida, 2002; Dumbacher *et al*., 2004; Williams, Brodie Jr & Brodie III, 2004; Opitz & Müller, 2009; Saporito *et al*., 2012; Savitzky *et al*., 2012). Animals capable of synthesizing their own toxins would not be expected to differ geographically in levels of chemical defense unless there was genetic variation among populations in the ability to synthesize toxins or geographic variation in their suite of predators (Thompson, 2005). However, geographic variation in chemical defense would be more likely to exist among animals dependent on prey for sequestered chemical defenses if critical prey species are distributed unevenly or have geographically variable toxicity (Hanifin *et al*., 1999; Thompson, 2005).

**Figure 1:**
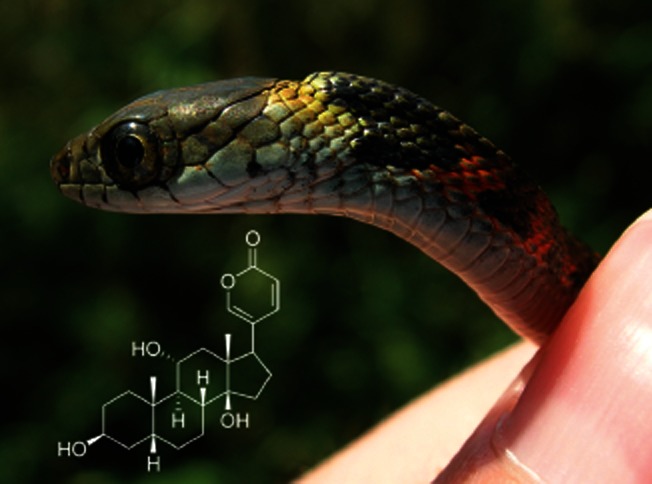
Juvenile *Rhabdophis tigrinus* on Ishima and one of the 17 major bufadienolides (gamabufotalin, inset) that we have identified in the nuchal gland fluid of this species. Gamabufotalin is also found in the skin secretion of toads, among many other bufadienolides. Note the prominent nuchal ridge on the dorsal surface of the neck, reflecting the large underlying nuchal glands.

*Rhabdophis tigrinus* is an oviparous Asian snake (Colubridae: Natricinae) that possesses unusual defensive glands on the neck known as nuchal glands (Fig. 1; Nakamura, 1935; Smith, 1938; Mori *et al*., 2012). The contents of the nuchal glands have been shown to contain toxic bufadienolides (Akizawa *et al*., 1985*a*,*b*), which irritate mucous membranes and cause corneal injuries (Kawashima, 1959; Suzuki, 1960; Asahi *et al*., 1985). Bufadienolides are cardiotonic steroids, similar to cardiac glycosides in foxglove plants (*Digitalis*), that act by inhibiting the sodium–potassium pump, causing arrhythmia and cardiac failure in high doses (Melero, Medarde & San Feliciano, 2000). The bufadienolides in the nuchal glands of *R. tigrinus* are similar to those found in the skin secretions of toads (Bufonidae; Erspamer, 1994), which are often consumed by *R. tigrinus* (Mori & Moriguchi, 1988). Although toads are capable of synthesizing their defensive bufadienolides from cholesterol (Siperstein, Murray & Titus, 1957; Porto, Baralle & Gros, 1972), *R. tigrinus* is dependent on dietary toads from which it can sequester these compounds for storage in the nuchal glands (Hutchinson *et al*., 2007). Bufadienolides sequestered from toads can be provisioned by female *R. tigrinus* to offspring *in utero* by deposition of those compounds in yolk and by transfer to oviducal eggs during gestation (Hutchinson *et al*., 2008, 2012).

The majority of reported natural predators of *R. tigrinus* are birds, but fish, giant salamanders, raccoon dogs (*Nyctereutes*) and other snakes also have been reported to prey on this species (Tanaka & Mori, 2000). Field observations of encounters between *R. tigrinus* and its predators are lacking, but presumably, contact between the irritating nuchal gland fluid and the eyes or mouth of a bird or mammal serves as an effective deterrent (Asahi *et al*., 1985; Mori *et al*., 2012). *Rhabdophis tigrinus* has been shown in laboratory experiments to exhibit specific defensive behaviors that orient the nuchal glands toward a perceived threat (Mori & Burghardt, 2001), demonstrating the defensive function of the nuchal glands.

*Rhabdophis tigrinus* primarily consumes anurans (frogs), including the Japanese toad *Bufo japonicus* (Fukada, 1959; Moriguchi, 1982; Mori & Moriguchi, 1988). Unfed hatchlings of *R. tigrinus* are at least twice as likely to feed on toads or nonbufonid frogs than fish, demonstrating an innate preference for anurans (Mori *et al*., 2012). However, toads are not entirely sympatric with *R. tigrinus*, so some populations of snakes lack access to toads as prey. Within Japan, *R. tigrinus* occurs on the main islands of Honshu, Shikoku and Kyushu, as well as some of the smaller Japanese islands (Nakamura & Uéno, 1963; Toriba & Sawai, 1990; Takeuchi *et al*., 2012).

**Figure 2:**
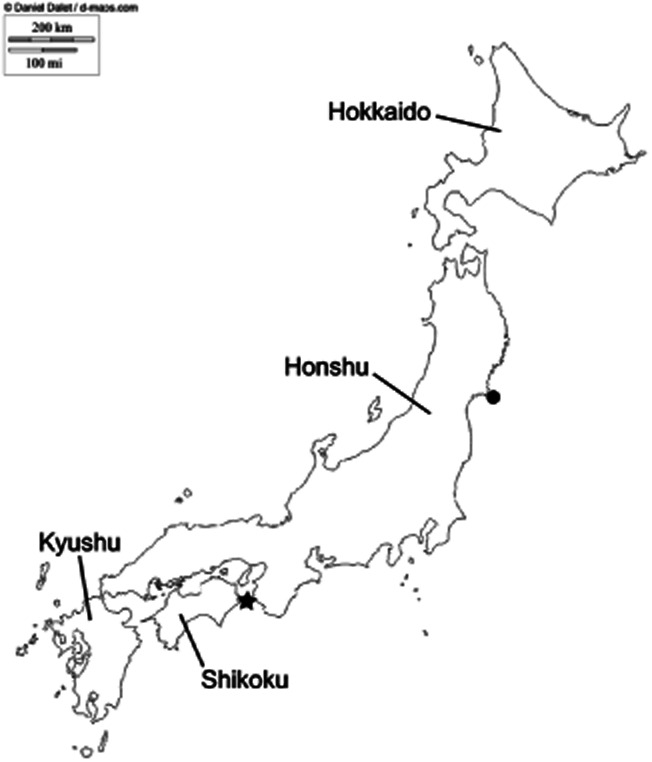
Map of Japan showing the locations of Kinkasan Island, Miyagi Prefecture (black circle; 38°17.7′N, 141°34.0′E) and Ishima Island, Tokushima Prefecture (black star; 33°50.9′N, 134°48.8′E). Base map obtained from http://d-maps.com/carte.php?lib=japan_map&num_car=4467&lang=en.

In this study, we analyzed the chemistry of the nuchal gland fluid of *R. tigrinus* from two small Japanese islands: Ishima (Tokushima Prefecture), where toads are abundant, and Kinkasan (Miyagi Prefecture), where toads are absent (Fig. 2). Ishima supports dense populations of anurans, including *B. japonicus* (Bufonidae), *Hyla japonica* (Hylidae), *Glandirana rugosa* (formerly *Rana rugosa*; Ranidae) and *Pelophylax nigromaculatus* (formerly *Rana nigromaculata*; Ranidae). In contrast, the only frog present on Kinkasan is the nonbufonid *Rana tagoi* (Ranidae; Nagata & Mori, 2003).

Our primary aim for this study was to compare the bufadienolide content of *R. tigrinus* from Kinkasan and Ishima. Substantial differences would presumably reflect disparities in defensive efficacy of the nuchal glands between these populations. Furthermore, we aimed to establish experimentally the effects of diet on the defensive chemistry of *R. tigrinus* from these two islands.

## Methods

### Experimental design and sample collection

For the feeding study, 12 gravid female *R. tigrinus* were collected on Kinkasan Island (38°17.7′N, 141°34.0′E) and Ishima Island (33°50.9′N, 134°48.8′E), six from each location. They were transported to Kyoto University, where they were fed only fish and nonbufonid frogs that lack bufadienolides (*P. nigromaculatus*). Following oviposition, the eggs were incubated between 25 and 30°C. The hatchlings were reared on controlled diets of either exclusively nonbufonid prey or a combination of toads and nonbufonid prey. The nonbufonid prey items consisted primarily of fish (*Oryzias latipes*; Adrianichthyidae) and frogs (*H. japonica*); salamanders (*Hynobius tokyoensis* and *Hynobius lichenatus*; Hynobiidae) were used as supplemental food for individuals that lived longer than 6 months. The toads used as prey were metamorphic (newly metamorphosed) *B. japonicus* from Niijima Island, Tokyo Prefecture. Most hatchling snakes were sacrificed after 7–9 months and frozen for later collection and analysis of nuchal gland fluid.

For chemical analyses, samples of metamorphic toads and nuchal gland fluid of snakes were prepared. Two frozen metamorphic toads (0.12 g each) were cut into pieces and extracted individually in methanol for chemical analyses. From each previously frozen snake, we expressed all nuchal glands onto a portion of Kimwipe (Kimwipes Wipers S-200; Kimberly-Clark, Dallas, TX, USA) while wearing nitrile, polyethylene or latex gloves; each Kimwipe was then placed into a vial of methanol with forceps and covered with a Teflon-lined cap. The forceps were rinsed in methanol from a glass pipette and were dried with a Kimwipe between each individual; gloves were also changed between individuals. At least one control vial consisting of a Kimwipe in methanol was prepared at the end of each sampling session to test for cross-contamination between samples. At least six hatchlings from each of 12 clutches were sampled for a total of 51 hatchlings from Ishima (age 5–280 days) and 48 hatchlings from Kinkasan (age 9–223 days). All samples were stored at −20°C.

To determine quantities of bufadienolides in wild-caught *R. tigrinus*, we collected nuchal gland fluid from individuals on toad-free Kinkasan Island and toad-rich Ishima Island. We sampled six wild-caught adults (two females and four males) from Ishima in summer and early fall and six wild-caught adults (three females and three males) from Kinkasan in summer. Additionally, we analyzed nuchal gland fluid from 20 wild-caught juveniles (10 females and 10 males) that were sampled on Ishima in September. The juveniles ranged in snout–vent length (SVL) from 166 to 292 mm, suggesting that they had hatched that year (Fukada, 1992). The majority of these animals were captured, sampled and released; those that were taken into captivity were sampled prior to feeding or were fed only nonbufonid prey. A partial sample of nuchal gland fluid was collected from four of the six adults from Kinkasan (some nuchal glands were left intact) so the animals could be used for subsequent studies; we have preliminary evidence to suggest that nuchal glands do not regenerate after use (A. H. Savitzky & A. Mori, pers. obs.).

To assess the chemical profiles of some of the prey available to *R. tigrinus* on Ishima and Kinkasan, we sampled parotoid gland secretion from toads (*B. japonicus*) on Ishima and skin secretion from *R. tagoi* on Kinkasan. The parotoid glands of toads can be squeezed to collect bufadienolide-rich secretions on Kimwipes, but *Rana* spp. lack parotoid glands. Therefore, we used a transcutaneous amphibian stimulator (Grant & Land, 2002) to collect skin secretions from *R. tagoi*. The frogs were stimulated and then sections of Kimwipes were rubbed over their backs and submerged in individual vials of methanol. Our animal use protocols conformed to institutional policies and practices.

### Chemical analyses

Methanolic extracts of prey species and nuchal gland fluid from snakes were evaporated to dryness, reconstituted and analyzed by proton nuclear magnetic resonance (^1^H-NMR) spectroscopy and/or high-performance liquid chromatography (HPLC). The whole-body extracts of two metamorphic *B. japonicus* from Niijima Island (the population used in the feeding experiment), as well as parotoid gland secretion from adult toads on Ishima, were prepared and analyzed by nuclear magnetic resonance (NMR) spectroscopy and HPLC. The presence or absence of bufadienolides was determined by analyzing unfractionated samples on Unity INOVA 400-, 500-, or 600-MHz NMR spectrometers (Varian, Palo Alto, CA, USA) equipped with Oxford magnets (Oxford Instruments, Eynsham, Witney, Oxon, UK) and 5-mm inverse-detection hydrogen, carbon and nitrogen (HCN) or dual broadband gradient (DBG) probes.

To obtain quantitative data, we analyzed nuchal gland fluid from 99 hatchlings in the feeding experiment, as well as 20 wild-caught juveniles and 6 wild-caught adult *R. tigrinus* from Ishima. The samples were reconstituted in known volumes of methanol (typically 0.3 mL for samples with small quantities of bufadienolides or no bufadienolides, and 1.5–4.2 mL for concentrated samples) for analysis with HPLC. Most samples were analyzed with an Agilent (Santa Clara, CA, USA) 1100 series HPLC equipped with a quaternary pump, diode array detector and automated sampler. A reversed-phase 250 × 10 mm Supelco (Bellefonte, PA, USA) Discovery® HS C18 column was used for most samples (3.4 mL min^−1^ and 25 *μ*L injection volume), but several samples were analyzed with a Varian Pursuit XRs 3 *μ*m 250 × 4.6 mm C18 column at 0.75 mL min^−1^ (5 or 10 *μ*L injection volume). A solvent gradient using a mixture of methanol and water was used. The methanol was held at 20% (v/v) for 2 min and then increased linearly over 38 min to 100%. After 2 min at 100% methanol, the mixture was returned to 20:80 methanol:water.

Several samples were analyzed using a Waters 2790 HPLC (Waters Corporation, Milford, MA, USA) with an internal microtiter plate autosampler and Waters 2487 dual wavelength detector set to 225 and 300 nm, which approximate the absorbance maxima of bufadienolides. We used a Dionex Acclaim 120 C18 5 *μ*m 4.6 × 150 mm column (Thermo Scientific, Sunnyvale, CA, USA) with a flow rate of 1 mL min^−1^ with this HPLC.

To calculate the total quantity of bufadienolides present in each sample, we calibrated the HPLC using a series of dilutions of telocinobufagin (T. Spande and J. W. Daly) and cinobufotalin (Sigma C1147-10 mg; Sigma-Aldrich, St. Louis, MO, USA). The equations obtained from the linear trend lines fitted to the dilution curves were used to convert the cumulative area in mAU⋅s (milliabsorbance units⋅seconds) of bufadienolide peaks at 280 nm into milligrams of bufadienolides. Peaks produced by bufadienolides were identified by their characteristic UV-absorbance spectra, and only those peaks with areas greater than 15.0 mAU⋅s were used to calculate the total quantity of bufadienolides in each sample.

To identify individual bufadienolides, we compared chromatograms of the samples to those from standards containing 17 previously identified compounds from a related feeding experiment (electronic supplementary material, Supporting Information Fig. S1b; Hutchinson *et al*., 2007). The standards were reanalyzed with HPLC to determine any changes in retention time before comparing the two sets of data. Of the 17 bufadienolides we have identified in *R. tigrinus*, compounds **6** and **7** coelute, so they could not be distinguished from one another using HPLC. The software used to analyze the chromatograms was ChemStation for LC3D Rev. A.09.01 (Agilent).

### Statistics

To determine the effect of the total mass of toads consumed on the quantity of bufadienolides in nuchal gland fluid, we calculated Spearman's rank correlation coefficients for hatchling *R. tigrinus* from Kinkasan and Ishima. We used SPSS version 11.0 (Chicago, IL, USA) for statistical analyses and plotted data using SigmaPlot version 9.0 (SYSTAT, San Jose, CA, USA).

We conducted an analysis of covariance (ANCOVA) to test for the effect of sex on the quantity of bufadienolides in 20 wild-caught, juvenile *R. tigrinus* from Ishima. We used sex as the fixed factor and mass and SVL as covariates. To test for the homogeneity of slopes, we ran a preliminary ANCOVA including interaction terms of sex⋅mass and sex⋅SVL. Both interaction terms were nonsignificant (sex⋅mass *P* = 0.361; sex⋅SVL *P* = 0.273), so the interaction terms were removed and the ANCOVA was rerun without them (Engqvist, 2005). The assumption of equality of variances was met (Levene's test, *P* = 0.435), as was the assumption of normality (Shapiro–Wilk test, *P* = 0.674 for females, *P* = 0.425 for males).

## Results

### Prey items

**Figure 3:**
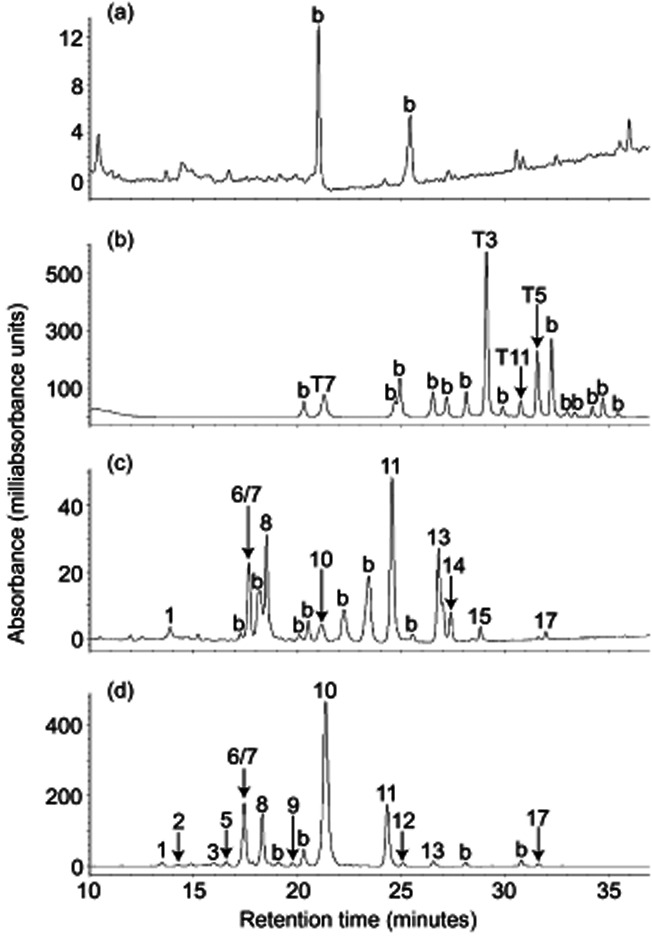
HPLC chromatograms of (a) a pooled sample of whole-body extracts of two metamorphic toads (*Bufo japonicus*) from Niijima Island, which was the population used in the feeding experiment, (b) parotoid gland secretion from an adult *B. japonicus* from Ishima Island, (c) nuchal gland fluid of a toad-fed hatchling *Rhabdophis tigrinus* from toad-free Kinkasan Island, (d) nuchal gland fluid of a wild-caught juvenile *R. tigrinus* on toad-rich Ishima Island. Numbers above peaks correspond to identified bufadienolides (see electronic supplementary material, Supporting Information Fig. S1, for structures of compounds). Peaks from unidentified bufadienolides are indicated by ‘b’. The differences in elution patterns of bufadienolides between toads and snakes reflect modifications of sequestered compounds by the snakes, as reported previously (Hutchinson *et al*., 2012).

Adult and metamorphic toads (*B. japonicus*) possessed bufadienolides (Fig. 3a,b), whereas the skin secretion of *R. tagoi* lacked those compounds. Whole-body extracts of metamorphic *B. japonicus*, which were fed to the hatchling *R. tigrinus* in the experimental group, contained small quantities of bufadienolides, as confirmed by NMR spectroscopy and HPLC (Fig. 3a). In a pooled sample of two whole-body extracts of metamorphic toads, each of which weighed 0.12 g, the approximate total quantity of bufadienolides was 0.02 mg. However, these metamorphs were not homogenized prior to extraction, so this amount of bufadienolides is probably an underestimate. The parotoid gland secretion of adult *B. japonicus* from Ishima contained large amounts of bufadienolides (Fig. 3b; electronic supplementary material, Supporting Information Fig. S1a).

### *R. tigrinus* from Kinkasan

**Figure 4:**
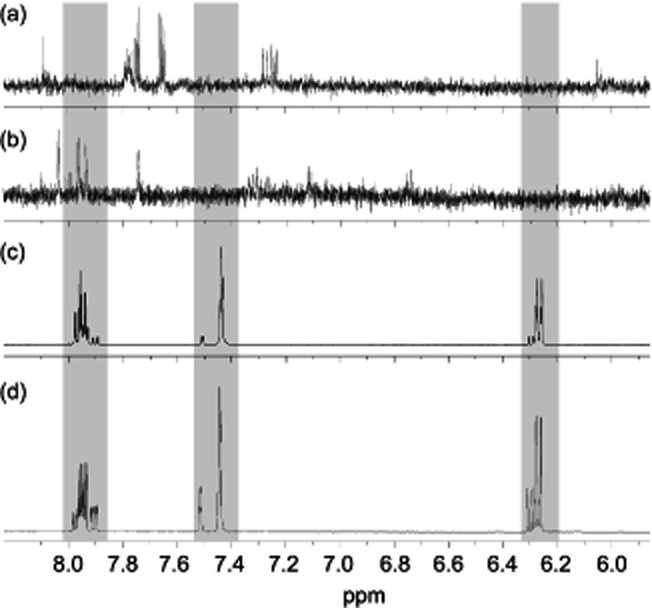
Aromatic region of ^1^H-NMR spectra from the nuchal gland fluid of *Rhabdophis tigrinus*. The gray bars highlight the three regions diagnostic of bufadienolides. (a) Fish-fed hatchling from toad-free Kinkasan Island, (b) adult from Kinkasan Island, (c) fish-fed hatchling from Ishima, (d) adult from Ishima. Bufadienolides are absent in (a) and (b); bufadienolides are present in large quantities in (c) and (d), as indicated by peaks in each of the highlighted regions.

**Figure 5:**
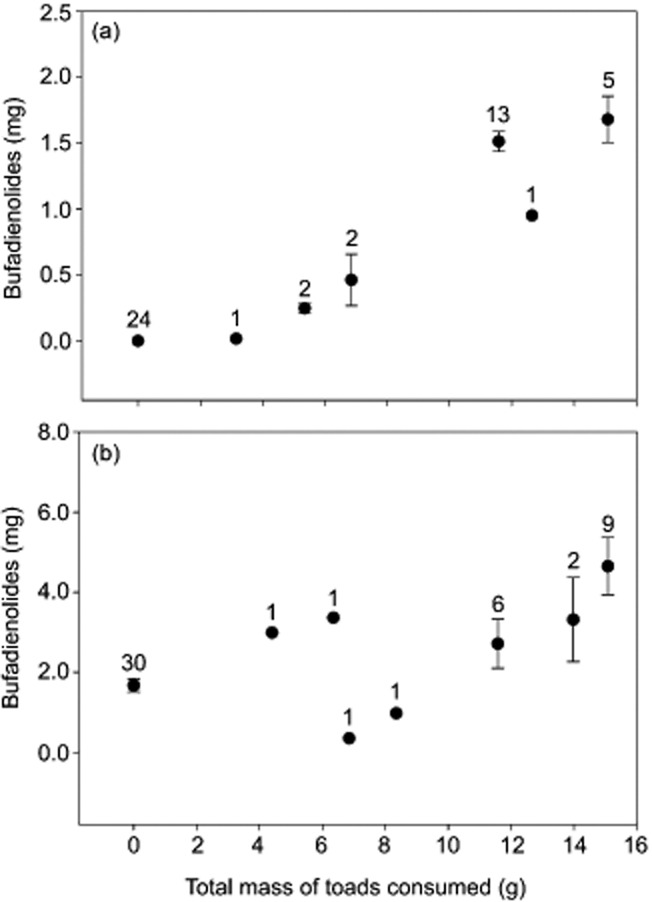
Total mass of toads consumed (g) versus quantity of bufadienolides (mg) in nuchal gland fluid of hatchling *Rhabdophis tigrinus* from (a) toad-free Kinkasan Island (Spearman's rho = 0.947, *P* < 0.0005) and (b) toad-rich Ishima Island (Spearman's rho = 0.559, *P* < 0.0005). Numbers of replicates are indicated above data points or error bars.

All six adult *R. tigrinus* from toad-free Kinkasan Island (three females and three males) lacked bufadienolides in their nuchal gland fluid (Fig. 4b), whereas hatchlings produced variable results depending on the individual's diet in captivity. Unfed and non-toad-fed hatchlings lacked bufadienolides (Fig. 4a), whereas hatchlings that were fed metamorphic toads (*B. japonicus*) possessed such compounds (Fig. 3c). Toad-fed hatchlings from all six clutches sequestered at least eight bufadienolides from their bufonid prey, with compound **11** being the most abundant bufadienolide in each sample (Table 1). The quantity of bufadienolides in the nuchal gland fluid increased as the mass of toads consumed increased (Fig. 5a; Spearman's rho = 0.947, *P* < 0.0005).

**Table 1:** Bufadienolides in juvenile *Rhabdophis tigrinus* from toad-free Kinkasan Island, fed controlled diets

	Clutch 1 (*N* = 12)	Clutch 2 (*N* = 17)	Clutch 3 (*N* = 14)	Clutch 4 (*N* = 18)	Clutch 5 (*N* = 13)	Clutch 6 (*N* = 15)
Unfed or non-toad-fed	None (*n* = 4)	None (*n* = 5)	None (*n* = 4)	None (*n* = 4)	None (*n* = 4)	None (*n* = 3)
Toad-fed	1, 2, 4, 6/7, 8, 9, 10, 11, 13, 14, 15, 17 (*n* = 4)	1, 2, 3, 4, 5, 6/7, 8, 9, 10, 11, 13, 14, 15, 17 (*n* = 4)	1, 2, 3, 4, 5, 6/7, 8, 10, 11, 13, 14, 15, 17 (*n* = 4)	1, 6/7, 8, 10, 11, 13, 14, 15 (*n* = 4)	1, 2, 3, 5, 6/7, 8, 10, 11, 13, 14, 15, 17 (*n* = 4)	1, 2, 3, 4, 5, 6/7, 8, 10, 11, 13, 14, 15 (*n* = 4)

*N* represents the number of hatchlings per clutch, whereas *n* indicates the number of individuals analyzed. Bufadienolides present in the majority of individuals per group are identified by compound numbers (see electronic supplementary material, Supporting Information Fig. S1b, for structures of compounds). The bufadienolide present in the largest quantity per group is underlined.

### *R. tigrinus* from Ishima

A strikingly different pattern emerged from the analysis of hatchling *R. tigrinus* from Ishima Island, which harbors a dense population of toads (*B. japonicus*). The unfed and non-toad-fed hatchlings typically possessed large quantities of bufadienolides (Fig. 4c). As an example, three unfed hatchlings from one clutch each contained 1.4–2.0 mg of bufadienolides in their nuchal gland fluid. Most hatchlings that were fed toads also contained large quantities of bufadienolides (Fig. 5b). Similar to the pattern seen in toad-fed hatchlings from Kinkasan, *R. tigrinus* from Ishima increased the quantity of bufadienolides in their nuchal gland fluid as the mass of toads consumed increased (Fig. 5b; Spearman's rho = 0.559, *P* < 0.0005).

Most unfed and non-toad-fed hatchlings from Ishima possessed seven or more bufadienolide compounds in their nuchal gland fluid (Table 2). Hatchlings that were fed toads from Niijima Island contained additional bufadienolides that were absent in their non-toad-fed counterparts (Table 2). Compound **14** was found only in those hatchlings that had been fed toads in captivity (Table 2).

**Table 2:** Bufadienolides in juvenile *Rhabdophis tigrinus* from toad-rich Ishima Island, fed controlled diets

	Clutch 7 (*N* = 21)	Clutch 8 (*N* = 7)	Clutch 9 (*N* = 14)	Clutch 10 (*N* = 14)	Clutch 11 (*N* = 18)	Clutch 12 (*N* = 43)
Unfed and non-toad-fed	1, 2, 3, 5, 6/7, 8, 9, 10, 11 (*n* = 4)	2, 3, 5, 6/7, 8, 9, 10, 11, 12, 13, 17 (*n* = 4)	2, 3, 5, 6/7, 8, 9, 10, 11, 13, 17 (*n* = 3)	3, 5, 6/7, 8, 9, 10, 11 (*n* = 4)	2, 3, 5, 6/7, 8, 9, 10, 11, 17 (*n* = 8)	1, 2, 3, 5, 6/7, 8, 9, 10, 11, 12, 13, 17 (*n* = 7)
Toad-fed	2, 3, 5, 6/7, 8, 9, 10, 11, 13, 14, 17 (*n* = 4)	1, 2, 3, 5, 6/7, 8, 9, 10, 11, 13, 14, 15 (*n* = 2)	2, 3, 5, 6/7, 8, 9, 10, 11, 13, 14, 17 (*n* = 4)	3, 5, 6/7, 8, 9, 10, 11, 13, 14, 15, 17 (*n* = 4)	1, 2, 3, 5, 6/7, 8, 9, 10, 11, 12, 13, 14, 17 (*n* = 3)	1, 2, 3, 5, 6/7, 8, 9, 10, 11, 12, 13, 14, 17 (*n* = 4)

*N* represents the number of hatchlings per clutch; *n* indicates the number of individuals analyzed. Bufadienolides present in the majority of individuals per group are identified by compound numbers (see electronic supplementary material, Supporting Information Fig. S1b, for structures of compounds). The bufadienolide present in the largest quantity in the majority of individuals per group is underlined. Two bufadienolides were present in equally large quantities in toad-fed individuals in clutch 10.

The six wild-caught adults from Ishima each possessed considerable quantities of bufadienolides in their nuchal gland fluid (Fig. 4d), and males contained extremely large amounts. Four adult males weighing 53–152 g possessed 27.0–68.9 mg of bufadienolides (mean = 43.7 mg). Nuchal gland fluid of two females weighing 275 and 652 g contained 7.3 and 20.4 mg of bufadienolides, respectively. Males had 0.34–0.69 mg of bufadienolides per gram of body mass (mean = 0.48 mg g^−1^), whereas both females had 0.03 mg g^−1^. This difference reflects both the larger body size of females and the smaller absolute quantity of bufadienolides that they possess relative to males.

**Figure 6:**
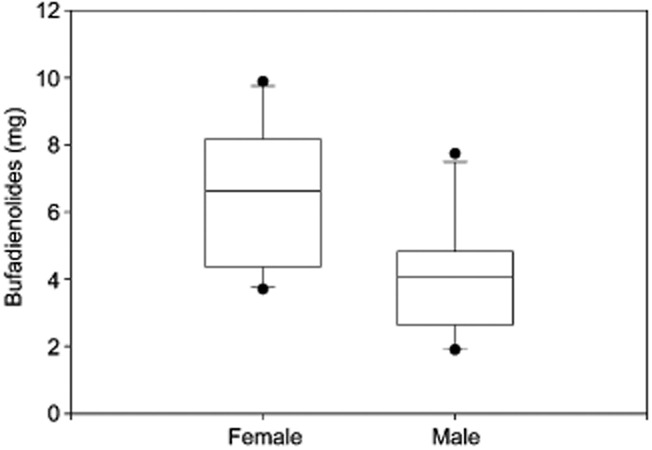
Quantities of bufadienolides (mg) in nuchal gland fluid of wild-caught female and male juvenile *Rhabdophis tigrinus* from toad-rich Ishima Island (ANCOVA *F* = 6.849; d.f. = 1,16; *P* = 0.019). Boxes represent the 25th and 75th percentiles and the line within each box indicates the median value. Outliers are located near the ends of the error bars.

The analysis of 20 wild-caught juvenile *R. tigrinus* on Ishima revealed large amounts of bufadienolides in both sexes (Fig. 3d), with females possessing significantly larger quantities than males. Females possessed an average of 6.5 mg of bufadienolides in their nuchal glands (range: 3.7–9.9 mg; *n* = 10), whereas males harbored an average of 4.1 mg (range: 1.9–7.8 mg; *n* = 10; Fig. 6). An ANCOVA revealed a significant difference between females and males in their quantities of bufadienolides (*F* = 6.849; d.f. = 1,16; *P* = 0.019). The covariates of mass and SVL did not affect bufadienolide quantity (*P* = 0.325 and *P* = 0.234, respectively). Compounds **1**–**13** and **17** were identified in this group of snakes, and all 20 individuals contained compounds **6**/**7** or **10** (gamabufotalin) in the greatest quantity, as did the hatchlings from Ishima that were used in the feeding experiment (Table 2).

## Discussion

Our results demonstrate clearly that the quantity of bufadienolides present in the nuchal glands of *R. tigrinus* reflects the availability of toads in the environment and, in captivity, is influenced by the diet of hatchlings. Hatchlings from toad-free Kinkasan Island lack bufadienolides in their nuchal glands at hatching because their dams (mothers) did not have access to toads as prey, from which the females could have sequestered and provisioned defensive bufadienolides to their offspring. These hatchlings lack bufadienolides after being fed nonbufonid frogs, fish and/or salamanders. However, hatchlings from Kinkasan are capable of sequestering bufadienolides in their nuchal glands when fed toads in the laboratory, and the quantity of bufadienolides they possess increases significantly with the mass of toads consumed.

Analysis of six adult *R. tigrinus* from Kinkasan demonstrated that the adults from this island also lack bufadienolides in their nuchal glands, which provides further evidence that this species is incapable of synthesizing defensive bufadienolides, even as adults. Therefore, the nuchal glands of *R. tigrinus* from Kinkasan are likely ineffective at deterring predators, which explains why snakes from this island tend to flee rather than use their nuchal glands in defensive displays (Mori & Burghardt, 2000). However, because these snakes have retained the ability to sequester toxins from bufonid prey, as demonstrated by our feeding experiment, they could regain the use of their nuchal glands for defense in the event of an introduction of toads to Kinkasan.

In contrast, all hatchling *R. tigrinus* from Ishima, where toads are abundant, possessed bufadienolides in their nuchal gland fluid, regardless of diet. Presumably, these compounds in unfed individuals and those fed nonbufonid prey were provisioned to them by their dams *in utero*, although the dams were not available for chemical sampling in this study. However, in support of this hypothesis, six adults from Ishima collected on subsequent expeditions possessed large quantities of bufadienolides. We have demonstrated previously that female *R. tigrinus* produce offspring with quantities of bufadienolides reflecting bufadienolide levels in the dams (Hutchinson *et al*., 2008) and that females can provision bufadienolides through deposition in yolk and by transfer to oviducal eggs during gestation (Hutchinson *et al*., 2008, 2012). In most cases, toad-fed hatchlings possessed larger quantities of bufadienolides than non-toad-fed hatchlings. The toad-fed hatchlings contained a greater diversity of bufadienolides than their non-toad-fed siblings, and the compounds found only in toad-fed snakes were those sequestered directly from the diet and not maternally provisioned. The difference between the most abundant bufadienolide in hatchlings from Ishima (compound **6**/**7** or **10**) and that in toad-fed hatchlings from Kinkasan (compound **11**) may reflect geographic or ontogenetic variation in the chemical profiles of *B. japonicus*, or physiological differences in sequestration abilities between *R. tigrinus* from Kinkasan and Ishima.

Most hatchlings from Ishima that were fed nonbufonid diets were sacrificed at 7–9 months of age, and their nuchal gland fluid was sampled after freezing. Thus, in most cases, the quantity of bufadienolides for these experimental hatchlings represents maternally provisioned compounds that were retained by the juveniles for more than 6 months, which is a pattern that we have reported previously for *R. tigrinus* (Hutchinson *et al*., 2008). It is not known how long these compounds ultimately persist in the snakes, but it is likely that the offspring must ingest toads at some point during their lives, if only to counter dilution of maternally provisioned toxins as they grow.

The 20 juvenile, wild-caught *R. tigrinus* from Ishima all possessed large quantities of bufadienolides in their nuchal gland fluid, and females contained significantly larger quantities of these defensive compounds than males. This difference could have arisen due to unequal maternal provisioning between male and female offspring *in utero*, perhaps due to the ability of female embryos to take up greater quantities of bufadienolides than males. Alternatively, the females may have been more successful at obtaining bufonid prey after hatching. However, *R. tigrinus* hatchlings emerge from their eggs in August to mid-September (Fukada, 1959), and we sampled these juveniles in September, so they had not had much time to forage.

Regardless of the mechanism that results in juvenile females possessing larger quantities of bufadienolides than juvenile males, the larger quantity of defensive compounds in females could prove important to their fitness. It is possible that females mobilize bufadienolides stored in their nuchal glands during vitellogenesis (yolk production) and/or during gestation to provide bufadienolides to their offspring prior to hatching. The nuchal glands contain a dense network of capillaries (Hutchinson *et al*., 2007), which likely serves to deliver bufadienolides from the oral mucosa or digestive tract to the nuchal glands. Those vessels may also carry bufadienolides from the nuchal glands to the liver (for deposition in yolk during vitellogenesis) and/or the oviducts for delivery to developing embryos. Thus, females would require larger quantities of bufadienolides than males so they can defend both themselves and their offspring from predators.

Although juvenile females from Ishima possess larger quantities of bufadienolides than juvenile males, our limited sample of adults from that island suggests that adult male *R. tigrinus* possess larger amounts of bufadienolides than adult females. On the basis of body mass, the bufadienolide content of adult males exceeds that of females by an order of magnitude. This is surprising because females reach a much larger body size than males (Fukada, 1992), so a larger proportion of toads in the population are available to females of this gape-limited predator. It is possible that the two adult females from Ishima possessed a lower quantity of bufadienolides than expected because they had transferred the toxins from their nuchal glands to their eggs, although this hypothesis remains untested. Further research is needed to explain why females possess more bufadienolides than males as juveniles, but the opposite is true for adults.

The presence of bufadienolides in the nuchal gland fluid of *R. tigrinus* from Ishima and the absence of those compounds in snakes from Kinkasan may strongly affect the fitness of individuals on these two islands. The large quantity of bufadienolides found in the nuchal gland fluid of most individuals from Ishima may result in enlarged nuchal ridges (Fig. 1), more effective defensive displays involving the glands and greater levels of chemical defense. *Rhabdophis tigrinus* from Kinkasan typically flee from perceived predatory threats (Mori & Burghardt, 2000), which is likely due to the lack of sequestered dietary compounds in that population. Thus, it is possible that *R. tigrinus* on toad-rich Ishima Island are better defended against their predators than are individuals on the toad-free island of Kinkasan.

## Supporting Information

**Figure S1** Major bufadienolides identified from the parotoid gland secretion of *Bufo japonicus* (a) and the nuchal gland fluid of *Rhabdophis tigrinus* (b). For structure elucidation of the shown compounds, see Hutchinson *et al*. (2007, 2008). Note the corrected chain length of **T11** (Hutchinson *et al*. 2008). Compound **8** is 11*α*-hydroxytelocinobufagin, compound **10** is gamabufotalin, compound **13** is hellebrigenin, and compound **17** is telocinobufagenin. With kind permission from Springer Science and Business Media: Hutchinson, D.A., Savitzky, A.H., Mori, A., Burghardt, G.M., Meinwald, J. & Schroeder, F.C. (2012). Chemical investigations of defensive steroid sequestration by the Asian snake *Rhabdophis tigrinus*. *Chemoecology* **22**, 199–206, fig. 1, © Springer Basel AG 2011.

## References

[b1] Akizawa T, Yasuhara T, Azuma H, Nakajima T (1985a). Chemical structures and biological activities of bufodienolides in the nucho-dorsal glands of Japanese snake, *Rhabdophis tigrinus*. J. Pharmacobiodyn.

[b2] Akizawa T, Yasuhara T, Kano R, Nakajima T (1985b). Novel polyhydroxylated cardiac steroids in the nuchal glands of the snake, *Rhabdophis tigrinus*. Biomed. Res.

[b3] Asahi H, Kohtari Y, Chiba K, Mishima A (1985). Effect of the nucho-dorsal gland venom of the yamakagashi snake on the eye. Folia Ophthalmol. Jpn.

[b4] Daly JW (1995). The chemistry of poisons in amphibian skin. Proc. Natl. Acad. Sci. USA.

[b5] Dumbacher JP, Wako A, Derrickson SR, Samuelson A, Spande TF, Daly JW (2004). Melyrid beetles (*Choresine*): a putative source for the batrachotoxin alkaloids found in poison-dart frogs and toxic passerine birds. Proc. Natl. Acad. Sci. USA.

[b6] Eisner T, Eisner M, Siegler M (2005). Secret weapons: defenses of insects, spiders, scorpions, and other many-legged creatures.

[b7] Engqvist L (2005). The mistreatment of covariate interaction terms in linear model analyses of behavioural and evolutionary ecology studies. Anim. Behav.

[b8] Erspamer V, Heatwole H, Barthalmus GT, Heatwole AY (1994). Bioactive secretions of the amphibian integument. Amphibian biology.

[b9] Fukada H (1959). Biological studies on the snakes. V. Food habits in the fields. Bull. Kyoto Gakugei Univ., B.

[b10] Fukada H (1992). Snake life history in Kyoto.

[b11] González A, Hare JF, Eisner T (1999). Chemical egg defense in *Photuris* firefly “femmes fatales.”. Chemoecology.

[b12] Grant JB, Land B (2002). Transcutaneous amphibian stimulator (TAS): a device for collection of amphibian skin secretions. Herpetol. Rev.

[b13] Hanifin CT, Yotsu-Yamashita M, Yasumoto T, Brodie ED, Brodie ED (1999). Toxicity of dangerous prey: variation of tetrodotoxin levels within and among populations of the newt *Taricha granulosa*. J. Chem. Ecol.

[b14] Hutchinson DA, Mori A, Savitzky AH, Burghardt GM, Wu X, Meinwald J, Schroeder FC (2007). Dietary sequestration of defensive steroids in nuchal glands of the Asian snake *Rhabdophis tigrinus*. Proc. Natl. Acad. Sci. USA.

[b16] Hutchinson DA, Savitzky AH, Mori A, Meinwald J, Schroeder FC (2008). Maternal provisioning of sequestered defensive steroids by the Asian snake *Rhabdophis tigrinus*. Chemoecology.

[b15] Hutchinson DA, Savitzky AH, Mori A, Burghardt GM, Meinwald J, Schroeder FC (2012). Chemical investigations of defensive steroid sequestration by the Asian snake *Rhabdophis tigrinus*. Chemoecology.

[b17] Kawashima J (1959). Disturbance of the eye by snake venom (*Natrix tigrina*) II. J. Rev. Clin. Ophthalmol.

[b18] Melero CP, Medarde M, San Feliciano A (2000). A short review on cardiotonic steroids and their aminoguanidine analogues. Molecules.

[b19] Mori A, Burghardt GM (2000). Does prey matter? Geographic variation in antipredator responses of hatchlings of a Japanese natricine snake (*Rhabdophis tigrinus*. J. Comp. Psychol.

[b20] Mori A, Burghardt GM (2001). Temperature effects on anti-predator behaviour in *Rhabdophis tigrinus*, a snake with toxic nuchal glands. Ethology.

[b22] Mori A, Moriguchi H (1988). Food habits of snakes in Japan: a critical review. Snake.

[b21] Mori A, Burghardt GM, Savitzky AH, Roberts KA, Hutchinson DA, Goris RC (2012). Nuchal glands: a novel defensive system in snakes. Chemoecology.

[b23] Moriguchi H (1982). Appearance, movements and food habits of snakes at Minasegawa, Kanagawa, Japan. Snake.

[b24] Nagata E, Mori A (2003). A record of *Elaphe conspicillata* from Kinkazan Island, Miyagi Prefecture. Bull. Herpetol. Soc. Jpn.

[b25] Nakamura K (1935). On a new integumental poison gland found in the nuchal region of a snake, *Natrix tigrina*. Mem. Coll. Sci. Kyoto Imperial Univ.

[b26] Nakamura K, Uéno SI (1963). Japanese reptiles and amphibians in colour.

[b27] Nishida R (2002). Sequestration of defensive substances from plants by Lepidoptera. Annu. Rev. Entomol.

[b28] Opitz SEW, Müller C (2009). Plant chemistry and insect sequestration. Chemoecology.

[b29] Porto AM, Baralle FE, Gros EG (1972). Biosynthesis of bufadienolides in toads III – experiments with [2-^14^C]mevalonic acid, [20-^14^C]3*β*-hydroxy-5-pregnen-20-one and [20-^14^C]cholesterol. J. Steroid Biochem.

[b30] Saporito RA, Donnelly MA, Spande TF, Garraffo HM (2012). A review of chemical ecology in poison frogs. Chemoecology.

[b31] Savitzky AH, Mori A, Hutchinson DA, Saporito RA, Burghardt GM, Lillywhite HB, Meinwald J (2012). Sequestered defensive toxins in tetrapod vertebrates: principles, patterns, and prospects for future studies. Chemoecology.

[b32] Siperstein MD, Murray AW, Titus E (1957). Biosynthesis of cardiotonic sterols from cholesterol in the toad, *Bufo marinus*. Arch. Biochem. Biophys.

[b33] Smith MA (1938). The nucho-dorsal glands of snakes. Proc. Zool. Soc. Lond. B.

[b34] Suzuki R (1960). Disturbance of the eye by snake venoms (*Natrix tigrina* (Boie)). J. Clin. Ophthal.

[b35] Takeuchi H, Ota H, Oh H-S, Hikida T (2012). Extensive genetic divergence in the East Asian natricine snake, *Rhabdophis tigrinus* (Serpentes: Colubridae), with special reference to prominent geographical differentiation of the mitochondrial cytochrome *b* gene in Japanese populations. Biol. J. Linn. Soc.

[b36] Tanaka K, Mori A (2000). Literature survey on predators of snakes in Japan. Curr. Herpetol.

[b37] Thompson JN (2005). The geographic mosaic of coevolution.

[b38] Toriba M, Sawai Y, Gopalakrishnakone P, Chou LM (1990). Venomous snakes of medical importance in Japan. Snakes of medical importance (Asia-Pacific Region).

[b39] Williams BL, Brodie ED, Brodie ED (2004). A resistant predator and its toxic prey: persistence of newt toxin leads to poisonous (not venomous) snakes. J. Chem. Ecol.

